# Integrated continuous bioprocessing: Economic, operational, and environmental feasibility for clinical and commercial antibody manufacture

**DOI:** 10.1002/btpr.2492

**Published:** 2017-06-02

**Authors:** James Pollock, Jon Coffman, Sa V. Ho, Suzanne S. Farid

**Affiliations:** ^1^ Dept. of Biochemical Engineering University College London Gordon Street London WC1H 0AH UK; ^2^ Pfizer Biotherapeutic Pharmaceutical Sciences 1 Burtt Road Andover MA; ^3^ Present address: Merck Manufacturing Division, Merck & Co., Inc. NJ USA; ^4^ Present address: Boehringer Ingelheim Fremont, Inc CA USA

**Keywords:** antibody manufacture, fed‐batch, perfusion culture, continuous chromatography, process economics

## Abstract

This paper presents a systems approach to evaluating the potential of integrated continuous bioprocessing for monoclonal antibody (mAb) manufacture across a product's lifecycle from preclinical to commercial manufacture. The economic, operational, and environmental feasibility of alternative continuous manufacturing strategies were evaluated holistically using a prototype UCL decisional tool that integrated process economics, discrete‐event simulation, environmental impact analysis, operational risk analysis, and multiattribute decision‐making. The case study focused on comparing whole bioprocesses that used either batch, continuous or a hybrid combination of batch and continuous technologies for cell culture, capture chromatography, and polishing chromatography steps. The cost of goods per gram (COG/g), E‐factor, and operational risk scores of each strategy were established across a matrix of scenarios with differing combinations of clinical development phase and company portfolio size. The tool outputs predict that the optimal strategy for early phase production and small/medium‐sized companies is the integrated continuous strategy (alternating tangential flow filtration (ATF) perfusion, continuous capture, continuous polishing). However, the top ranking strategy changes for commercial production and companies with large portfolios to the hybrid strategy with fed‐batch culture, continuous capture and batch polishing from a COG/g perspective. The multiattribute decision‐making analysis highlighted that if the operational feasibility was considered more important than the economic benefits, the hybrid strategy would be preferred for all company scales. Further considerations outside the scope of this work include the process development costs required to adopt continuous processing. © 2017 The Authors Biotechnology Progress published by Wiley Periodicals, Inc. on behalf of American Institute of Chemical Engineers *Biotechnol. Prog.*, 33:854–866, 2017

## Introduction

A major challenge facing the biopharmaceutical industry centers on how best to improve research and development (R&D) productivity while reducing R&D and manufacturing costs.^1^, ^2^, ^3^, ^4^ Identification of strategies to reduce nonclinical R&D costs can yield significant improvements in R&D productivity given that they typically represent 20–30% of R&D costs.^5^, ^6^ Clinical manufacturing costs and process validation batches account for a significant proportion of the nonclinical R&D costs. Hence, biopharmaceutical engineers and scientists are keen to explore ways to develop more cost‐effective and agile manufacturing processes while continuing to meet product quality targets. Given the lengthy, complex, and highly regulated nature of the development pathway for biopharmaceutical drugs, the industry has been debating which biopharmaceutical production technologies will facilitate industrialization of the sector.^7^, ^8^, ^9^, ^10^

In recent years, continuous bioprocessing has seen a resurgence of interest related to its potential to challenge the established position of the batch platform for the production of biopharmaceuticals such as monoclonal antibodies (mAbs). As a result, several companies have been evaluating continuous bioprocessing technologies^11^, ^12^, ^13^, ^14^ to determine if they can realize their potential benefits such as higher equipment utilization rates, smaller facility footprints, reduced cycle times, lower investment costs, and lower production costs. Recent FDA strategic plans^15^ and Quality by Design (QbD) initiatives appear to encourage such explorations of continuous biomanufacture.

A holistic assessment of continuous bioprocessing requires evaluation of the potential of integrated continuous bioprocesses across multiple products moving through the development pathway. This paper aims to achieve this by developing and applying a prototype decisional tool to address the following topical questions. Is there a business case for continuous bioprocessing for early phase manufacture? How does the business case change for commercial multiproduct manufacture? Will tomorrow's process be a hybrid of batch and continuous technologies? This study builds upon previous studies at UCL^16^, ^17^, ^18^ that explored the batch versus continuous question initially for upstream processing and then for capture chromatography. This paper extends the analysis to integrated continuous bioprocesses with consideration of the impact of both development phase and company portfolio size. The insights from the decisional tool will be illustrated through the use of industrial case studies that provide economic, environmental and operational perspectives on the decision to select batch versus continuous processes for clinical and commercial mAb manufacture.

### Background on perfusion culture and continuous chromatography in bioprocessing

#### Perfusion Culture

Fed‐batch cell culture has been established as the platform choice for most mAbs in recent years given the increase in fed‐batch titres combined with their ease of operation.^7^, ^19^, ^20^ Historically, the use of perfusion culture systems has been hampered by greater logistical and validation complexity as well as higher likelihoods of technical failures.^16^, ^21^, ^22^ However, more recent perfusion culture retention devices aim to overcome some of these obstacles with the promise of higher productivities, lower failure rates, and the ability to link to single‐use bioreactors.^16^ The recent performance trajectory of perfusion culture systems has prompted renewed interest in their potential.

Pollock et al.^16^ provide a summary of the perfusion culture systems utilized by 12 commercial therapeutic biologics that include recombinant blood factors, enzymes and mAbs. Filtration‐based retention devices in perfusion culture have experienced a technology evolution from internal spin‐filters to external spin‐filters and more recently to tangential flow filtration (TFF) or alternating tangential flow filtration (ATF) (Repligen Corporation, Waltham, MA) in an effort to minimize filter fouling and avoid premature culture termination.^23^ These more recent retention devices are being employed in the commercial production of mAbs (e.g., Simponi^®^ and Stelara^®^, Janssen Biotech).^16^ The ATF system achieves media exchange by circulating the broth back and forth between the bioreactor and an external hollow‐fiber filter via the action of a diaphragm pump.^24^, ^25^ As a result, the ATF perfusion system enables reduced filter fouling instances and consequences and higher cell densities and hence productivities compared to earlier perfusion systems.

On the process economics front, there are few contributions that examine the economic feasibility of filtration‐based perfusion culture systems. Lim et al.^21^, ^22^ compared a spin‐filter perfusion to a fed‐batch strategy under uncertainty, for a mAb output of 50 kg/year and a titre of 1 g/L. The authors used a stochastic analysis to demonstrate the reduction required in the failure rate of the spin‐filter perfusion strategy for it to compete with the fed‐batch strategy at this scale. Pollock et al.^16^ presented a cost of goods comparison of fed‐batch strategies to first generation (spin‐filter) and second generation (alternating tangential flow filtration, ATF) perfusion systems for the commercial manufacture of mAbs in dedicated facilities. The case study explores the impact of a number of key factors: (a) single‐use bioreactors and scale of production on costs, (b) equipment failure rates on robustness, and (c) qualitative concerns (e.g., ease of development) on the technology rankings. The authors illustrate how the economic competitiveness of whole bioprocesses employing perfusion culture processes depends on the cell density increase achievable with perfusion and the equipment failure rates compared to fed‐batch strategies. The tool's predictions that the spin‐filter perfusion strategy struggles to compete on economic, environmental, operational, and robustness fronts at most titres and scales provides insight into its limited use in industrial processes. In contrast the ATF perfusion strategy was predicted to offer economic benefits that outweigh its lower robustness for cases with peak cell densities that are at least threefold higher than in fed‐batch for scenarios of commercial production in dedicated facilities.

#### Semicontinuous Chromatography

The current and future mAb purification platforms rely on a series of orthogonal chromatography steps with Protein A as the preferred primary capture step.^8^, ^19^, ^20^, ^26^ Protein A resins represent the leading material cost contributor in most platforms.^16^, ^17^ For manufacture of early phase material, Protein A resin can account for 50% of the direct costs; however, during commercial manufacture, it contributes typically to 10% of the direct costs.^17^ During clinical manufacture, product‐specific chromatography resins are often used for just a few cycles and then discarded particularly if the drug candidate is unsuccessful. As a result the resins do not realize their full potential cycle lifetime. This is a particular concern in mAb development and improving utilization of these expensive resins can have a significant effect on the (pre)clinical manufacturing costs by reducing the cost burden associated with failed drug candidates.

Semicontinuous chromatography has been shown to be an effective way to increase resin utilization and hence reduce resin volumes required by 40–50%, step buffer consumption by 40–50% and overall process buffer consumption by 12–15%.^11^, ^12^, ^16^ Different continuous chromatography technologies currently available from vendors include the periodic counter current (PCC) system (3–4 columns) from GE Healthcare (Uppsala, Sweden), BioSMB (6–12 columns) from Pall Life Sciences (Port Washington, NY), BioSC (2–6 columns) from Novasep (Pompey, France), and SMBC (4–8 columns) from Semba Biosciences (Madison, WI). These systems increase utilization by dividing a standard batch column into multiple portions. This allows the first column to be loaded to 100% breakthrough while redirecting the flowthrough onto the next column to capture the target. This makes use of what would usually be unsaturated capacity in a standard single‐column chromatography system. Several biopharmaceutical manufacturers have been actively evaluating these technologies. For example, GE's PCC system has been reported to be evaluated by Amgen,^27^ Centocor/Janssen,^28^ Genentech,^11^ Genzyme,^12^, ^13^ and Pfizer/UCL,^17^ while Pall's (previously Tarpon's) BioSMB system has been reported to be investigated by Bayer,^29^ Biogen‐Idec,^30^ Merck,^14^ and Pfizer.^31^

Financial implications of adopting continuous chromatography have been presented by Pollock et al.^17^ The study explores the potential of continuous capture chromatography to reduce clinical and commercial mAb manufacturing costs. The technology evaluation integrated small‐scale experimentation, which was verified with a continuous system in operation, with simulation assessment. The authors illustrate that whole bioprocesses that utilize continuous chromatography for product capture have the ability to offer significant direct cost savings in early clinical phase material generation; this can have a large impact considering the high clinical attrition rates.

#### Integrated Continuous Bioprocesses

Recent papers have detailed successful implementation of integrated, closed, and continuous bioprocesses linking an alternating tangential flow perfusion process to two semicontinuous chromatography steps for capture and polishing.^12^, ^13^ Results have been illustrated for the production of both stable (mAb) and less stable (enzyme) proteins in an uninterrupted manner over extended periods with consistent time‐based system performance and product quality. Such examples act as important proof‐of‐concept demonstrations for the sector of the potential of integrated continuous bioprocesses.

This paper scopes out a vision for a number of integrated continuous manufacturing processes and utilizes a decisional tool to provide novel insights on the suitability of these future manufacturing strategies across a matrix of industry scenarios. The paper presents a framework for evaluating integrated continuous processes rather than stepwise comparisons carried out in previous work. The analysis is extended to derive and incorporate operational risk scores considering the adoption of continuous technologies. These scores are combined with the calculated economic and environmental performance metrics so as to give an overall manufacturing strategy ranking. The impact of both development phase and company size is explored on the optimal manufacturing strategy in terms of batch and continuous unit operations. In all scenarios, the models spanned the whole bioprocess from cell culture through to polishing so as to capture the full impact of any upstream or downstream choice on the overall process economics and the impact of multiproduct facilities explored at each phase of development.

## Methods

### Visualising an integrated continuous process

A key concept of an integrated continuous process is that continuous, steady‐state processing extends from the bioreactor to the final purification operation. However, this concept is currently not possible in biopharmaceutical manufacture due to the lack of suitable technology and strict regulatory requirements. Continuous perfusion bioreactors for example do generate a continuous stream of harvested cell culture fluid (HCCF), but they can only achieve this in a batch operation. First, the cell culture has to reach the desired steady‐state cell density to achieve a constant concentration of HCCF and then can only produce a continuous stream of HCCF for a defined period, before a new cell culture batch is required. This semicontinuous mode of operation is also found in the search for continuous downstream processing operations where chromatography systems that are described as continuous are capable of continual loading but only generate discrete elution pools of product. The challenge is further complicated by the stringent quality and regulatory requirements that dominate biopharmaceutical manufacture. The ability of the manufacturing process to demonstrate viral clearance is critical, with a mandatory inclusion of two dedicated viral clearance operations (viral inactivation and viral retention filtration). Both of these operations are currently achieved in a batch operation; for example, in a viral inactivation step the product stream is held at a low pH for a defined period of time, before processing continues. A further regulatory complication is batch traceability, a key area of debate surrounding continuous processing, with the principal concern being “how do you define a batch?.”

These factors highlight how continuous processing is not currently possible in biopharmaceutical manufacture. However, the use of semicontinuous unit operations can lead to a semicontinuous manufacturing process, potentially capturing some of the economic advantages seen in continuous processing. The upstream can be operated in a semicontinuous manner by using perfusion culture, which is fed and bled at a constant rate to generate a constant stream of HCCF when steady‐state cell density is achieved. The downstream is more complicated due to the number of orthogonal purification operations. The initial capture of the product from HCCF can be achieved in a continuous manner, using semicontinuous chromatography. The resulting process stream is now being created in discrete elution volumes, which can either be pooled into larger volumes or processed individually before moving to the subsequent purification steps. These subbatches can be processed in the conventional batch manner for the remaining purification steps or can be processed in a continuous manner.

**Figure 1:**
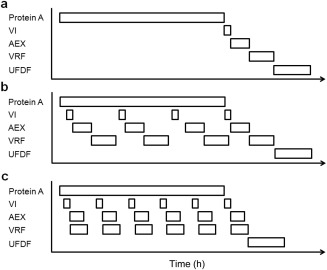
Downstream process scheduling for (a) the base case process sequence, (b) the continuous to batch process sequence, and (c) the continuous process sequence. Protein A, Protein A chromatography; VI, viral inactivation; AEX, anion exchange chromatography; VRF, viral retention filtration; UFDF, ultrafiltration/diafiltration.

Figure 1a shows the downstream scheduling for a typical process sequence operated in batch mode, where each step is completed before the product stream is passed to the next. Figure 1b shows an adapted process sequence where HCCF is continually loaded onto a semicontinuous chromatography step and the resulting elution volumes are pooled into larger volumes before proceeding in a batch manner similar to Figure 1a for the remaining purification steps. Figure 1c demonstrates a flow‐sheet that operates the anion‐exchange (AEX) chromatography step and virus retention filtration (VRF) step in a continuous manner. The product stream flows through the AEX chromatography column and straight into the VRF step. The VRF unit would be sized by calculating the filter area capable of matching the volumetric flowrate from the AEX chromatography step while maintaining the same transmembrane flowrate (20 LMH (Lm^−2 ^h^−1^)) seen in the batch processes (Figures 1a,b). Both process flowsheets operating the semicontinuous chromatography capture step collect all the processed subbatches (post VRF) into one final batch prior to the final diafiltration step. This pooling approach helps solve the regulatory requirement for batch traceability, by defining the batch as all the material created in one fermentation run. This approach also reduces the quality burden by reducing the number of batch releases for a given manufacturing strategy. Table 1 demonstrates how this concept results in five different manufacturing strategies, where the capture step is defined by the mode of Protein A chromatography used and the polishing steps (AEX and VRF) are defined by how the resulting purification steps are operated. The base case strategy employs a fed‐batch reactor generating a single discrete batch, which is purified in a batch manner (Figure 1a). Similarly the fed‐batch, continuous capture and batch polishing (FB‐CB) strategy also employs a fed‐batch reactor, but the ensuing batch is purified using semicontinuous chromatography in a 72 h window with the polishing steps operated in the batch manner (Figure 1b). In contrast, the ATF perfusion, continuous capture and batch polishing (ATF‐CB) strategy employs an alternating tangential flow (ATF) perfusion reactor to generate a constant stream of HCCF which is captured directly onto the semicontinuous chromatography step for the duration of the perfusion run, prior to batch operated polishing steps. The remaining strategies (FB‐CC; Fed‐batch, continuous capture, continuous polishing, and ATF‐CC; ATF perfusion, continuous capture, continuous polishing) both employ a continuous capture step that generates discrete subbatches, which are pooled and then processed in a continuous manner in the polishing purification steps as shown in Figure 1c.

**Table 1:** Mode of Operation for Key Stages of the Alternative Batch, Continuous, and Hybrid Manufacturing Strategies

Manufacturing Strategies	USP	Capture	Polishing
Base case	Fed‐batch	Batch	Batch
FB‐CB	Fed‐batch	Continuous	Batch
ATF‐CB	ATF perfusion	Continuous	Batch
FB‐CC	Fed‐batch	Continuous	Continuous
ATF‐CC	ATF perfusion	Continuous	Continuous

Note: USP refers to upstream processing.

### Decisional tool description

To successfully evaluate batch and semicontinuous biopharmaceutical unit operations, a decisional tool (Pollock et al., 2013a and 2013b)^16^, ^17^ was created that captured the operational, economic, environmental, and risk features associated with each strategy. The decisional tool integrated models on mass balancing, equipment sizing, bioprocess economics, scheduling, uncertainty analysis, and multiattribute decision‐making. The tool was built in a discrete‐event simulation environment (Extend v6, Imagine That! Inc., San Jose, CA). This permitted the dynamic consequences of resource constraints, delays, uncertainties, and equipment failures to be modeled in a temporal fashion and their impact on the key metrics to be computed. The tool was database‐driven (MySQL AB, Uppsala, Sweden) so as to facilitate the specification of processes and better manage the large input and output datasets required for multiple processes, uncertainty analysis and optimization. The tool incorporated specific features to capture the dynamics of continuous manufacture. These included scheduling features of perfusion culture related to interactions between the generation of daily perfusion harvests and the subsequent pooling and purification operations as well as the scheduling consequences of failure events occurring at random times during the perfusion cultures.^16^ The models for the continuous chromatography operations captured the experimentally derived design logic to generate the optimal system scale and operating parameters (e.g., switch time between columns).^17^

A precalculation and optimization module was created to run prior to the simulation of a particular scenario so as to assess all the possible equipment sizing strategies before selecting the optimum process configuration (e.g., 1x 6000 L versus 3x 2000 L bioreactors) for the given demand in terms of both production time and cost. Alongside equipment scaling the precalculation module also evaluated whether single‐use technologies could be implemented in the form of single‐use bioreactors and product holding bags, based on the process volumes generated and utilized by each unit operation.

The key economic metrics calculated by the tool were the capital investment and the cost of goods per gram (COG/g) for the complete manufacturing process (fermentation to bulk drug substance), as well as the manufacturing cost per launch (preclinical, clinical, process validation batches). The capital investment was determined using the Lang factor method as a function of the total equipment purchase cost. The COG/g comprised the annual direct (materials and labor) and indirect (depreciation and facility‐dependent overheads) operating costs divided by the annual product output as detailed in Farid et al.^32^ The key environmental metric calculated by the tool was the E‐factor,^33^ defined as the total mass of water or consumables used by the manufacturing strategy divided by the total mass of product. The tool's quantitative outputs were combined with qualitative operational metrics using a multiattribute decision‐making (MADM) technique,^34^ as described in the next section.

### Multiattribute decision‐making methodology

The weighted sum method provides a systematic mechanism for explicitly capturing qualitative and nonfinancial performance indicators in the evaluation. Its use has been illustrated in several fields including bioprocessing^16^, ^35^, ^36^ where alternatives were evaluated against a set of attributes that are typically considered intangible and conflicting. In this paper, the economic, environmental, and operational outputs were reconciled using the weighted sum method so as to identify the most attractive manufacturing strategy across a range of scenarios, with different weightings assigned to each of these categories. Table 2 lists all the attributes considered in the MADM analysis. The simulation tool was used to derive the economic and environmental attribute values. The attributes were ranked in order of importance, where a ranking of one indicates an attribute of greater significance. For example in the large‐sized company the cost per launch is more important than the commercial COG/g and the initial capital investment required in facility construction. In contrast the small‐sized company ranks the initial capital expenditure the highest, highlighting the differing financial philosophies found with company size. A large‐sized company's main aim is to reduce the cash outlay for a new drug and produce this material as cost‐effectively as possible. In contrast, the small‐sized company will have fewer resources to invest (facilities & drug development) and will therefore want to minimize these costs before looking to alternative funding sources upon product launch (licensing, partnerships, mergers, and acquisitions). The attributes representing the environmental feasibility were ranked equally because the environmental impact of water and consumable usage was deemed to be equally disadvantageous to the environment. The operational feasibility was represented by a risk score, which assesses the manufacturing strategies perceived robustness (likelihood of batch failure).

**Table 2:** Attribute Grouping and Weighting for Each Company Scale for the Multiattribute Decision‐Making

Attribute Group	Attribute Name	Large	Medium	Small
Economic feasibility	Manufacturing cost per launch	1	1	2
	Commercial COG/g	2	2	3
	Capital expenditure	3	2	1
Environmental feasibility	Water E‐factor	1	1	1
	Consumable E‐factor	1	1	1
Operational feasibility	Batch risk	1	1	1

Note: Rank of 1 refers to most important attribute and 3 to the lowest.

All attribute values were standardized^37^, ^38^ to convert them to a common dimensionless scale between 0 and 100. The relative importance of the total weighted economic, environmental, operational scores in the decision‐making process was captured using a set of combination ratios (dimensionless weight values) whose sum equals one.^37^ The overall aggregate strategy score $S_{j}$ is generated by the weighted sum method, using the following equation:

(1) $$S_{j}=\left|\frac{\sum_{i=1}^{n}r_{ijeconomic}}{n}\times R_{1}\right|+\left|\frac{\sum_{i=1}^{n}r_{ijenvironmental}}{n}\times R_{2}\right|+\left|\frac{\sum_{i=1}^{n}r_{ijoperational}}{n}\times R_{3}\right|$$

where *r_ijecononmic_*, *r_ijenvironmental_*, *r_ijoperationa_*_l_ represent the weighted scores for the economic, environmental and operational groups respectively (prior to combination) and $R_{1}$, $R_{2}$, and $R_{3}$ represent the economic, environmental, and operational combination ratios, respectively.

### Case study assumptions

The decision‐support framework was used to compare the cost‐effectiveness of the five alternative manufacturing strategies throughout the development pipeline for a range of company sizes, exploring the trade‐offs between reduced equipment scales versus increased manufacturing risk. Table 3 illustrates the clinical trials estimates used throughout this case study to calculate the amount of mAb required for each phase of the development pipeline. The earliest development phase captured in this case study is the Pre‐Clinical phase where material is required for nonprimate animal model studies. Assuming the average nonprimate (Macca Mulatta) body weight is ∼8 kg^39^ and the study includes 110 nonprimates (25% control group),^40^ a single 0.5 kg batch of mAb is required for the Pre‐Clinical development studies. The case study then uses the quick win, fail fast clinical development paradigm,^4^ where the material required for Phases I and II is generated in a single batch for the Proof‐of‐Concept (PoC) development phase. The average body weight of a US male was presumed to be 86 kg^41^ and therefore a single 4 kg batch of mAb would be required for PoC development also accounting for nonclinical uses. This amount increases to 40 kg of mAb for the phase III clinical trials and is produced by four 10 kg batches at the Commercial batch scale allowing parallel process validation studies. The 10 kg Commercial batch size is based on the median market demand of the top 15 mAb (200 kg)^8^ and the ability to process 20 batches per year in a typical fed‐batch scenario. The cell culture titre also increases with clinical phase, where due to continued process development the titre was assumed to increase twofold from the PoC batch to the Phase III and Commercial batches. The scenario produced a 2.5 g/L titre for the minimally developed Pre‐Clinical and PoC batch before increasing to a final titre of 5 g/L.

**Table 3:** Key Assumptions for the Alternative Batch, Continuous, and Hybrid Manufacturing Strategies

Variable	Values	
*Clinical Trial Estimates*		
Non‐human primate dosage (mg/kg body weight)	700	
Non‐human primate in Pre‐Clinical trial	100	
Patient dosage (mg/kg body weight)	7	
Number of doses per patient per year	26	
Individuals in Phase I clinical trials (single dose)	40	
Individuals in Phase II clinical trials (6 month dose)	200	
Individuals in Phase III clinical trials (year dose)	2,000	
*USP Process Parameters*	Fed‐batch	ATF
Cell culture time (days)	12	28
Harvest volumes	1	20
Max VCD (million cells/mL)	10	50
Max bioreactor volume (L)	20,000	1,500
Annual number of batches	20	10
*DSP Process Parameters*	Batch	PCC
Binding capacity (g/L)	40	65
Bed height (m)	0.25	0.1
Number of columns	1	3
Shift duration (hours)	12	24
*Cost Parameters*		
QCQA batch release costs ($/batch)	35,000	
Media cost ($/L)	3.1	
Protein A resin cost ($/L)	8,000	
AEX resin cost ($/L)	1,500	
Virus retention filtration membrane ($/m^2^)	3,250	
Labour cost ($/h)	58	
Chromatography process skid (15–600 L/h) ($)	226,000	
PCC process skid (15–600 L/h) ($)	1,080,000	
Chromatography column (Dia = 0.2 m) ($)	132,000	
Chromatography column (Dia = 2 m) ($)	218,000	

Note: USP refers to upstream processing, DSP to downstream processing.

The base case manufacturing strategy used in the case study was based on a generic two‐column mAb process.^7^, ^42^ The principal differences between the batch and semicontinuous unit operations are highlighted in Table 3. The key difference between the cell culture technologies is the length of culture, where a fed‐batch fermentation lasts 12 days allowing 20 batches to be processed a year from a single reactor. In contrast perfusion cell cultures can be run for much longer, however in this case study a culture duration of 28 days was selected, making an annual throughput of 10 batches possible. The 28‐day perfusion length was selected over longer lengths considering issues such as the time required for process validation or process performance qualification (PV/PPQ) batches as well as the ability to fit in more products per year. The perfusion‐based options were sized to yield the same kg outputs per phase with a view to keeping the scale constant between Phase III and Commercial stages as in the fed‐batch case. Hence, for example, Phase III or Commercial scenarios requiring greater than 10 × 10 kg perfusion batches per year would result in numbering up to multiple production lines. The semicontinuous PCC system utilizes three smaller columns compared to the batch system, these are loaded to 100% saturation increasing the binding capacity from 40 to 65 g of mAb per liter of resin. To achieve the higher productivity the PCC system must be operated continuously requiring a 24 h manufacturing shift.

Table 4 highlights the major differences between different sized companies with respect to the number of drug candidates (DC) at any given stage of the drug development pipeline. A large company has been defined as a company that aims to launch two new products per year. To achieve this level of success 20 new DC's must enter Pre‐Clinical trials, due to the high attrition rates seen in clinical development. The medium‐sized company aims to launch one product a year and therefore requires 10 DC's entering Pre‐Clinical trials per year. A small‐sized company that aims to launch a new product every 2 years requires only 5 DC's in Pre‐Clinical trials per year.

**Table 4:** Number of Drug Candidates Per Company Scale Scenario

Company Size	Pre‐Clinical	PoC	Phase III	Commercial
Large	20	14	4	2
Medium	10	7	2	1
Small	5	3	1	1*

*One successful launch every 2 years.

## Results and Discussion

The decision‐support framework was used to assess the cost‐effectiveness of five manufacturing strategies with different combinations of batch and continuous operations for cell culture, capture, and polishing steps throughout the drug development pipeline. This was initially carried out by determining the direct (labor, media, buffers, chromatographic resin, filter membranes, QCQA batch release costs, etc.) and indirect (depreciation and facility‐dependent overheads) costs. These were used to establish the cost of goods per gram across combinations of different development phases (Pre‐Clinical through to Commercial production) and company sizes (small, medium, and large). Each development phase required different manufacturing scales, batch numbers and material reuse strategies, and each company size resulted in different numbers of drug candidates at each development phase. The economic outputs were then considered alongside operational and environmental metrics using a multiattribute decision‐making technique for all the company sizes investigated.

### Impact of development phase on cost drivers

**Figure 2:**
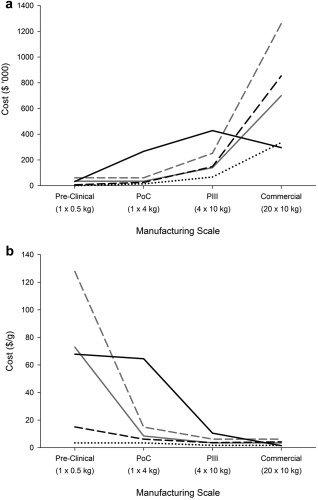
Direct cost of goods category breakdown across the different manufacturing scales required for each development phase for the base case scenario shown as (a) direct cost per product per phase and (b) direct cost per gram. Categories: labor costs (gray dashed line), QCQA batch release costs (gray solid line), chromatographic resin costs (black solid line), fermentation media (black dotted line), and single‐use components and buffers (black dashed line).

Figure 2 shows the individual cost components per product per phase as well as per gram for the base case batch scenario for each manufacturing scales in the development pipeline (0.5, 4, 40, and 200 kg) for a medium‐sized company. Figure 2a highlights that as expected the costs of chemicals (media, buffer) and single‐use components (e.g., filters, bags) increase per product across the development phases in proportion to the kg and batch output. Hence, the cost per gram for chemicals and single‐use components remain relatively constant in Figure 2b as is typical for variable costs. The resin costs also increase as the manufacturing scale increases across the development phases but in contrast to the other cost categories, the resin cost decreases at the Commercial scale of production. The requirement to keep the resins product‐specific, results in a high cost burden per batch in early development phases, because the resin is often discarded before reaching its full potential cycle lifetime. The early manufacturing scales (Pre‐Clinical and PoC) only use the resin to purify a single batch of material and therefore the resin purchase cost accounts for over 80% of the material costs per batch. The later manufacturing scales (Phase III and Commercial) use the resin to purify multiple batches of material and therefore reduce the cost impact of the expensive resin as reflected in Figure 2b. The resin accounts for approximately 67% of the Phase III material costs and 29% of the Commercial material costs. Both manufacturing scales utilize the same sized columns but only the Commercial scale uses the resin until its full lifetime and therefore the resin cost is spread over multiple batches and reduces the related resin costs shown.

**Figure 3:**
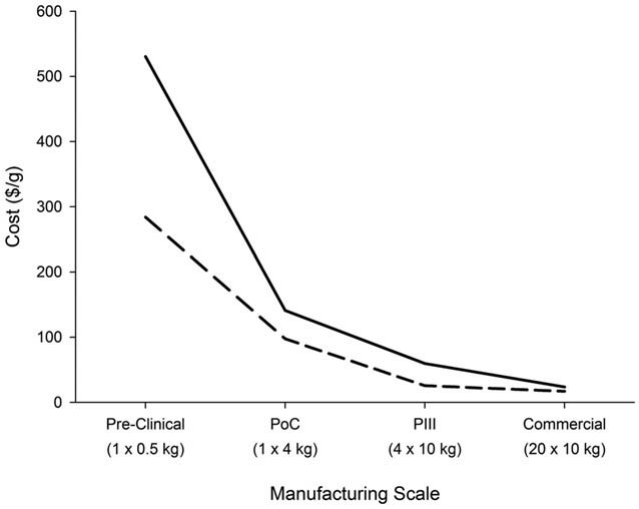
Direct (black dashed line) and indirect (black line) cost of goods per gram across the different manufacturing scales required for each development phase for the base case scenario.

The labor and QCQA costs are scale‐independent and rise in proportion to the increase in number of batches required per product across the development phases rather than the kg output (Figure 2a). As a result, their cost per gram values decrease with kg output (Figure 2b). Finally the indirect costs increase across the development phases in proportion to the increase in batch size and hence facility size. Figure 3 demonstrates as expected that the indirect cost per gram becomes less significant in the late clinical and Commercial phases since the costs are spread over more batches.

### Impact of company size on indirect costs

Table 4 highlighted the difference in drug candidate throughput for each company size throughout the development pipeline. The difference in drug candidate throughput will affect the utilization of the manufacturing suites and in turn impact the resulting manufacturing costs. Table 5 highlights the effect of company size on key indirect costs for the base case scenario at the PoC scale of manufacture. The capital expenditure required to construct the facility to generate a 4 kg PoC batch is the same for all the company sizes, however the resulting batch suite cost and indirect cost per gram is dependent on the batch throughput. The large‐sized company has the highest batch throughput with 14 drug candidates being processed a year resulting in an utilization rate of 70% and a batch suite cost of $292k. In contrast the small‐sized company has a batch throughput of three drug candidates resulting in a batch suite cost ($1,364k) that is 4.5‐fold higher relative to the large‐sized company.

**Table 5:** Effect of Company Size on Indirect Cost Per Gram for the Base Case Scenario at the PoC (4 kg) Manufacturing Scale

Company Size	Capital Expenditure (million $)	Batch Suite Cost ($/batch)	Indirect Cost/g ($/g)
Large	37.6	292,300	71
Medium	37.6	584,600	141
Small	37.6	1,364,000	330

### Batch versus continuous COG/g comparison

**Figure 4:**
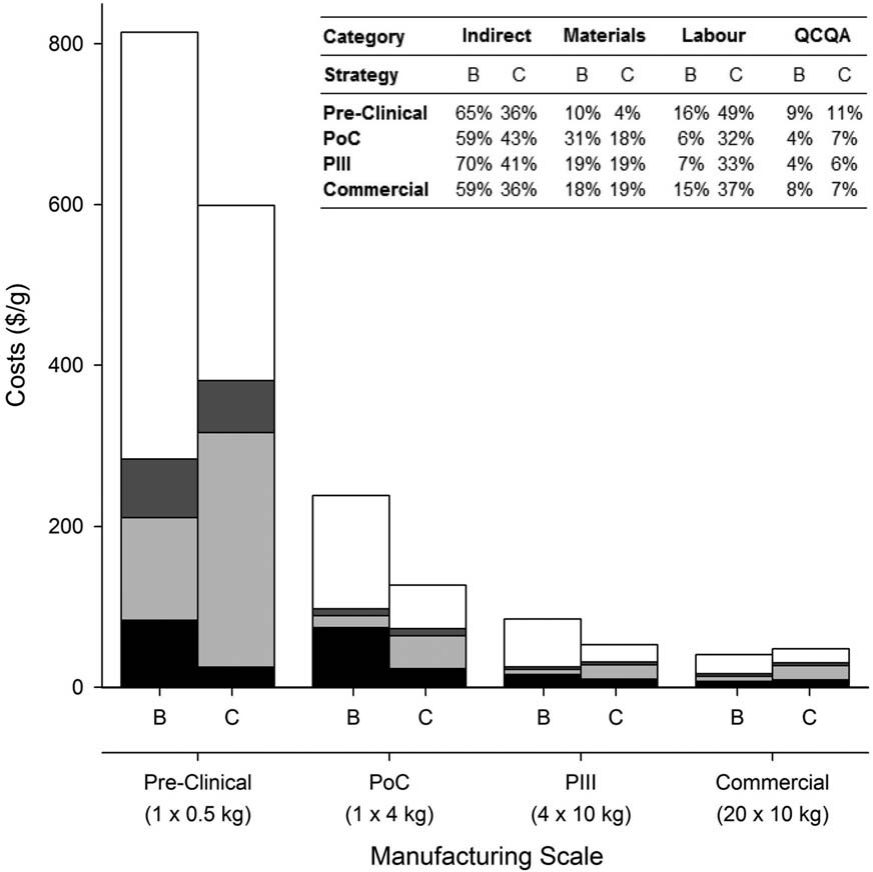
A comparison of the direct costs per gram for the base case batch strategy (B) and fully continuous strategy (C) on a category basis for material costs (black), labor costs (light gray), QCQA batch release costs (dark gray), and indirect costs (white), between the different manufacturing scales. The embedded table highlights the percentage cost contribution for the key direct cost categories.

Figure 4 shows the COG/g breakdowns for the base case batch strategy and fully continuous strategy (ATF‐CC) for the medium‐sized company. The analysis highlights that the Pre‐Clinical batch costs are dominated by indirect costs for the base case and direct costs for the continuous strategy. The larger equipment sizes seen in batch processing lead to a higher batch suite cost of $255K per batch versus $120K per batch for the smaller highly utilized continuous equipment. However, the continuous operation requires significant labor resources to support the continuous manufacturing operations that require a 24 h manufacturing shift and therefore this increases the overall direct costs.

The base case is still dominated by indirect costs in the later manufacturing scales in the development pipeline (PoC, Phase III, and Commercial). For example, the Phase III COG/g is dominated (70% of COG/g) by the batch suite cost (indirect costs), due to the costs only being spread over four batches in a commercial GMP facility. The continuous strategy sees a shift from the direct to indirect costs dominating COG/g for the later manufacturing scales. However, the labor costs still account for a third of the COG/g. Traditionally the direct costs are expected to dominate with an increase in kg output, however, due to the relatively low kg output in this scenario (200 kg) the indirect costs continue to dominate COG/g. The indirect COG/g does decrease in significance with increasing company size with direct costs starting to dominate as kg output increases (large company—2 × 200 kg products).

The smaller batch sizes seen earlier in the development pipeline have higher direct manufacturing costs per gram because some of the costs are scale independent. For example the QCQA batch release costs ($35,000) are constant between a Pre‐Clinical and Commercial batch, but due to the difference in kg output (0.5–200 kg) the batch strategies Pre‐Clinical QCQA cost per gram is $73/g compared to $3.4/g for the Commercial manufacturing scale. The same trend is seen in the batch strategy`s labour costs which accounts for nearly half the Pre‐Clinical direct manufacturing costs at $128/g compared to the Commercial manufacturing scale with labour costs of $6/g. The overall percentage of direct costs increases for the Commercial manufacturing of the batch strategy, even with the real decrease in direct costs per gram. This is due to the significant drop seen in batch suite costs, from the Phase III ($600K per batch) to Commercial ($240K per batch) manufacturing scale.

For the medium‐sized company, the tool outputs predict that the integrated continuous ATF‐CC strategy offers cost savings for Pre‐Clinical and Clinical production, but becomes less economically attractive at the Commercial scale. This is due to the requirement for a second manufacturing production line given the kg output per batch being matched to the Phase III requirement (10 kg) and maximum number of batches per year,^10^ resulting in the duplication of equipment (USP and DSP). The higher utilization rate of the GMP facility at this manufacturing scale is expected to lead to a significant reduction in indirect costs (as shown by the batch strategy). However, the extra equipment required for the additional production line means the reduction is not fully realized and with the significant labour requirements seen in the continuous strategy, the strategy is no longer able to offer an economically attractive COG/g as the batch scenario.

### Key economic metrics across company size and manufacturing scale

**Figure 5:**
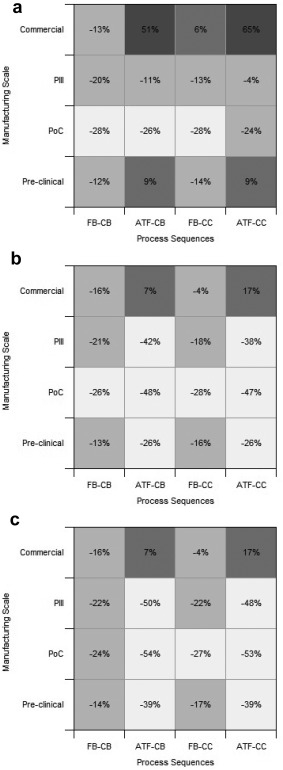
Contour plots showing the impact of manufacturing scale and manufacturing strategies on the percentage difference in cost of goods per gram relative to the base case scenario for (a) the large‐sized company, (b) the medium‐sized company, and (c) the small‐sized company. (Pre‐Clinical, 1 × 0.5 kg; PoC, 1 × 4 kg; Phase III, 4 × 10 kg; Commercial, 20 × 10 kg).

The impact of both manufacturing scale (Pre‐Clinical, PoC, Phase III, Commercial) and company size (small, medium, large) on the competiveness of the five alternative manufacturing strategies was investigated. The contour plots in Figures 5a–c show the percentage difference in cost of goods per gram relative to the base case strategy.

**Figure 6:**
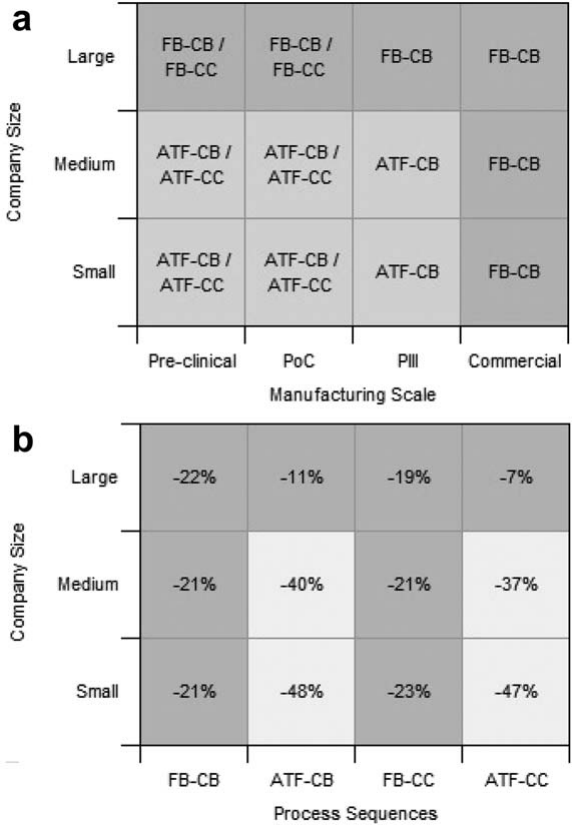
Contour plots showing the impact of manufacturing scale and manufacturing strategies on (a) the most economically attractive manufacturing strategies for each scenario and (b) the resulting manufacturing cost per launch for all company sizes relative to the base case manufacturing strategy. (Pre‐Clinical, 1 × 0.5 kg; PoC, 1 × 4 kg; Phase III, 4 × 10 kg; Commercial, 20 × 10 kg).

Figure 5 highlights that the ATF perfusion‐based manufacturing strategies are not able to compete with the fed‐batch strategies at the Commercial scale of manufacture regardless of company size. The difference is most pronounced for the large‐sized company (Figure 5a), where the high drug candidate throughput in the development pipeline, results in the need to manufacture two commercialized products in a year. The ATF perfusion strategies are only able to generate 10 batches per year per production line and therefore require four parallel perfusion reactors with dedicated purification trains to meet the 40 batch annual demand. The resulting facilities are approximately twice as expensive as the corresponding fed‐batch based facilities, which employ two staggered reactors utilising a single larger purification train. This effect is also seen for the medium and small‐sized companies (Figures 5b, 5c) where two production lines are required to manufacture 20 batches of a single successfully commercialized product. The resulting Commercial facilities are comparable in cost to the fed‐batch based facilities and offer the same level of capital expenditure saving (∼25%) versus the base case due to the use of a smaller purification train offered by the continuous capture step. Figures 5 and 6a highlight that the inability of the ATF perfusion strategies to utilize a single production line for Commercial manufacture, results in the fed‐batch based strategy FB‐CB being the most economically attractive Commercial manufacturing strategy for all company sizes.

**Figure 7:**
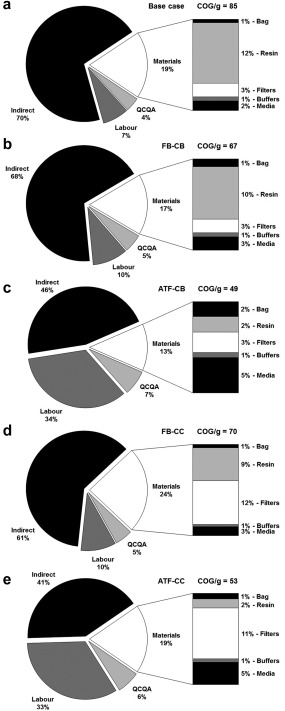
A comparison of cost of goods per gram with a detailed breakdown of material costs on a category basis for (a) the base case, (b) FB‐CB, (c) ATF‐CB, (d) FB‐CC, (e) ATF‐CC scenario for a Phase III clinical batch in a medium‐sized company.

Figure 5 highlights that the FB‐CB manufacturing strategy is the most consistent strategy, offering COG/g savings at all manufacturing and company scales relative to the base case. This is possible due to the continuous capture step which reduces the volume of expensive Protein A resin required and generates a more concentrated elution pool allowing a smaller purification train to be employed, reducing both direct and indirect batch costs. However, the FB‐CB does not offer the highest level of savings for product development stages, as highlighted in Figure 6a that shows the top ranking strategies across the different manufacturing and company scales. Instead, this is achieved by the ATF‐CB and ATF‐CC manufacturing strategy, depending on the phase of development. The ATF‐CB strategy offers superior COG/g savings during clinical manufacture because it is able to reduce the size of the purification train even further by the continuous generation of small volumes of HCCF. This has a significant impact on the dominant material costs by replacing the resin cost with media cost, due to the 10‐fold reduction in column volume and the fourfold increase in fermentation media use. The FB‐CC and ATF‐CC also reduce the scale of the purification train they employ, however, the continuous polishing steps result in the suboptimal scaling of the virus retention filtration operation. The batch‐operated polishing process strategies operate the virus retention filtration step for a complete 10 h shift, whereas continuous‐operated polishing process strategies run the operation for the shorter duration (2–3 h) of the preceding chromatographic step. This means a larger filter area is required per purification operation and when combined with the multiple subbatches processed per batch leads to a threefold increase in virus retention filtration costs, causing the filter costs to dominate the material costs. The increase in filter costs leads to a higher COG/g compared to the corresponding batch‐operated polishing manufacturing strategies, most noticeably for Commercial manufacture.

The manufacturing cost per launch of a successful drug candidate (DC) was a key economic metric used to compare the alternative manufacturing strategies encompassing the total risk‐adjusted clinical development manufacturing cost. The manufacturing cost per launch captures the costs of all the unsuccessful DCs incurred alongside the development of a successfully commercialized DC (10x Pre‐Clinical DCs, 7x PoC DCs, and 2x Phase III DCs). Figure 6b shows the percentage difference in the cost per launch of a successful drug candidate for all the alternative manufacturing strategies for all the company scales. The figure highlights how the FB‐CB strategy offers the biggest cost saving (22%) for a large‐sized company. In contrast the ATF‐CB or ATF‐CC strategy offers an even bigger cost saving for the small (∼40%) and medium‐sized (∼50%) companies, because only a single manufacturing line is required throughout the clinical phases of development. This allows a much smaller facility to be used compared to the FB‐CB strategy and as the company size decreases the cost contribution for the indirect costs increase, allowing the ATF‐CB/ATF‐CC strategy to have the cost per launch (small‐sized company).

Figure 7 presents a detailed breakdown of the COG/g for all the alternative manufacturing strategies in a medium‐sized company at the Phase III manufacturing scale. The figure highlights how the base case and alternative fed‐batch based manufacturing strategies COG/g are dominated by indirect costs, due to the larger fermentation and purification capabilities required compared to the smaller highly utilized ATF perfusion‐based strategies. The higher utilization of the smaller process sequences seen in the ATF perfusion‐based strategies is off‐set by the ∼2.5‐fold increase in labour demand required to operate the continuous capture step for the duration of the perfusion cell culture. The high labour demand and multiple production lines seen in the ATF perfusion‐based strategies explains the inability of the strategies to offer a competitive alternative to the base case for Pre‐Clinical manufacture in a large‐sized company.

### Multiattribute decision‐making

This section extends the analysis beyond economic metrics to include the environmental and operational benefits of each strategy.

#### Environmental Impact Analysis

The tool was also used to capture the water and consumable usage of the alternative manufacturing strategies to assess the environmental impact of the strategies across a range of manufacturing and company scales. E‐factor values were derived for the usage of water (cell culture media, process buffers, CIP buffers, and rinse water) and consumables (bags, membranes, and resins) within the manufacturing process. Typical mAb manufacturing strategies (base case) consume water from 3,000 to over 7,000 kg water per kilogram product. The cell culture steps consume between 20% and 25% of the total, with the chromatographic operations often surpassing 50% of the total.^43^

As expected, all the alternative manufacturing strategies have a lower water E‐Factor value in comparison to the base case strategy, where the difference in water usage can be directly related to the use of the continuous capture step and the resulting lower buffer requirement. Table 6 demonstrates how the ATF perfusion‐based manufacturing strategies have an even lower water E‐factor value than the fed‐batch based strategies. A typical ATF‐perfusion process without a continuous capture step has been shown to have higher process water usage compared to a fed‐batch process due to the high media usage (Pollock et al., 2013a).^16^ The removal of the dedicated primary clarification step and the use of the single‐use bioreactors (SUB) by the ATF perfusion process reduces the nonprocess water usage by ∼30% (Pollock et al., 2013a).^16^ These trends when combined with a continuous capture step allow the ATF perfusion‐based strategies to reduce their total water usage by 25–45%. In contrast the manufacturing strategies utilising continuous capture and polishing (FB‐CC, ATF‐CC) have higher water E‐Factor values compared to the continuous capture and batch polishing manufacturing strategies (FB‐CB, ATF‐CB). The increase in E‐Factor value is due to the higher number of subbatches processed every batch, causing an increase in CIP buffers and rinse water used in cleaning between subbatches.

**Table 6:** E‐factor Scores for the Alternative Batch, Continuous and Hybrid Manufacturing Strategies

Manufacturing Strategies	Water (kg/kg product)	Consumable (kg/kg product)
Base case	3,900–7,250	6–73
FB‐CB	3,000–6,400	8–61
ATF‐CB	2,150–5,500	6–35
FB‐CC	2,750–7,450	13–48
ATF‐CC	2,300–5,550	8–25

The consumable E‐Factor values are highly dependent on the amount of single‐use technologies and resin volume employed by the strategies. Table 6 highlights how all the manufacturing strategies have a lower consumable E‐Factor than the base case due to the use of the continuous capture step reducing the resin volumes used. The ATF perfusion‐based strategies have a lower E‐factor value than the fed‐batch based strategies even though they employ SUBs because this increase in consumable waste is countered by the 10‐fold reduction in resin volume seen.

#### Operational Risk Analysis

A risk score was assigned to each manufacturing strategy to assess the operational feasibility with respect to strategy robustness (likelihood of batch failure). The risk score is a ranking value used to compare the alternate strategies and does not capture a true value for batch failure risk. Table 7 shows the risk score for both the upstream and downstream sections of the manufacturing strategies. The upstream risk score was calculated by assuming that each addition to the bioreactor had a 1 in 1,000 chance of causing contamination (Pollock et al., 2013b).^17^ The fed‐batch based strategies have a total of ten reactor additions (initial media fill and nine feeds) and therefore have a 1% risk score. In contrast the perfusion strategies had approximately 28 additions due to the daily media exchanges and therefore have a risk score of 2.8%. A similar approach was also used for the downstream risk score, where for every virus retention filtration (VRF) operation there was a 1 in a 1,000 chance of a filter or quality control failure. The same logic was applied to chromatographic operations where every cycle there was a 1 in 1,000 chance of a failure event of which 10% of these would lead to a batch failure. Table 7 demonstrates how the ATF perfusion‐based strategies have a higher risk score for both the upstream and downstream due to the high number of media exchanges and cycles in the continuous chromatography capture step. The continuous capture and polishing based strategies also have high risk scores due to the numerous VRF operations, resulting in the ATF‐CC strategy having the highest risk score of all the strategies due to high number of processing operations per batch.

**Table 7:** Batch Operational Risk for the Alternative Batch, Continuous, and Hybrid Manufacturing Strategies

Manufacturing Strategies	USP Risk	DSP Risk	Overall Risk Score
Base case	1%	0.2%	1.2%
FB‐CB	1%	0.3%	1.3%
ATF‐CB	2.8%	2.3%	5.1%
FB‐CC	1%	1%	2%
ATF‐CC	2.8%	4.6%	7.4%

#### Overall Aggregate Strategy Scores

**Figure 8:**
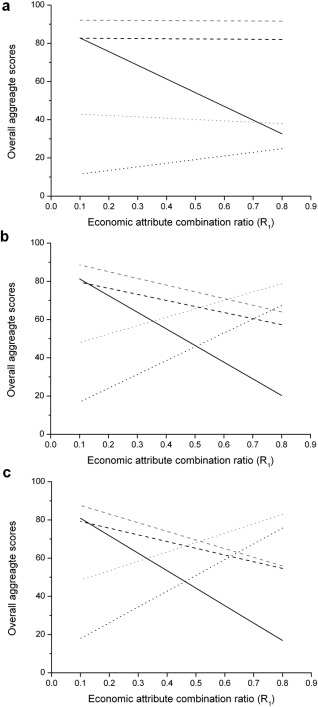
Sensitivity spider plots portraying the effect of the economic attribute combination ratio (*R*_1_) in the overall aggregate scores when the environmental combination rate is constant (0.1), for (a) the large‐sized company, (b) medium‐sized company, and (c) small‐sized company, for the base case (solid black line), FB‐CB (gray dashed line), ATF‐CB (gray dotted line), FB‐CC (black dashed line), and ATF‐CC (black dotted line). On the left‐hand side of each figure, when the economic attribute combination ratio is 0.1, the operational attribute combination ratio is 0.9 and operational benefits are considered more important in the overall aggregate score. On the right‐hand side of each figure, when the economic attribute combination ratio is 0.9, the operational attribute combination ratio is 0.1 and economic benefits are considered more important in the overall aggregate score.

This section discusses the results from deriving a single multiattribute score so as to reconcile any conflicting outputs. The impact of the relative importance of the economic, environmental, and operational scores on the ranking of the manufacturing strategies was explored by determining the overall aggregate strategy score for a range of combination ratios. Figure 8 illustrates the sensitivity of the overall aggregate strategy scores and hence rank order of the alternative manufacturing strategies to the economic and operational attribute combination ratios, for a range of company scales (large, medium, and small). In this particular scenario, the environmental attribute combination ratio was set to 0.1. Figure 8a illustrates that for a large‐sized company the FB‐CB is always the preferred manufacturing strategy regardless how important the economic or operational feasibility is ranked. The ATF perfusion‐based manufacturing strategies fail to achieve a high aggregate score due to their high risk scores and inability to offer significant economic advantage compared to the preferred FB‐CB manufacturing strategy. In a medium‐sized company (Figure 8b) when the economic benefits are twice as important as the operational benefits (*R*_1_ = 0.6, *R*_3_ = 0.3) the ATF‐CB and FB‐CB strategies are equally attractive. The ability of the ATF‐CB strategy to offer a superior ranking as the importance of the economic benefits increases is due to superior savings offered in the cost per launch and capital expenditure at this company scale. Figure 8c illustrates how the high importance placed on capital expenditure and cost per launch by the small‐sized company, results in the ATF‐CB becoming the preferred manufacturing choice with increasing economic importance. However, if the operational benefits are more important the FB‐CB strategy is still the favoured manufacturing strategy. When the operational attribute combination ratio is fixed at 0.1 and the environmental attribute combination ratio varied with the economic attribute combination ratio, the FB‐CC strategy is able to outcompete the FB‐CB strategy across all company scales, because the higher risk score is countered by the higher significance placed on the lower E‐factor ratings. The remaining relationships, with the ATF‐CB able to offer superior ranking as the importance of the economic benefits increases, are maintained for the medium and small‐sized companies.

## Conclusion

This paper has presented a feasibility evaluation of continuous biopharmaceutical manufacturing strategies utilising perfusion cell culture and semicontinuous chromatography throughout the product life cycle from Pre‐Clinical to Commercial manufacture. The decision‐support framework was configured to cope not only with individual continuous unit operations but also integrated continuous processes. The framework enabled a rigorous analysis of the feasibility of mAb manufacturing facilities based on the standard batch platform compared to alternative integrated and hybrid continuous manufacturing strategies across a range of manufacturing and company scales so as to represent scenarios of relevance to industry. The analysis determined the key cost contributors in each strategy, as well as the robustness via an operational risk score and environmental indices that act as useful E‐factor benchmarks for continuous processes. This enabled the economic, environmental and operational outputs to be assessed simultaneously. The tool predicts that the complete continuous strategy (ATF‐CC) may find it hard to compete on economic, environmental and robustness fronts for Commercial manufacture especially when multiple trains are required, but offers savings for material supply for product development stages (Pre‐Clinical, PoC, Phase III) due to the higher productivities and smaller footprint facilities. In contrast, the hybrid batch and continuous strategies (FB‐CB and ATF‐CB) outperform the continuous strategy for all manufacturing and company scales when weighing up multiple economic, operational and environmental perspectives. The FB‐CB strategy was shown to be the most consistent strategy offering savings at all manufacturing and company scales and is always the preferred manufacturing strategy for the large‐sized company. The ATF‐CB strategy is predicted to offer superior economic benefits for material supply during product development that outweigh its lower robustness and increased Commercial manufacturing costs for the medium and small sized companies as demonstrated in the multi‐attribute analysis. However, the analysis highlighted that if the operational feasibility is considered more important than the economic benefits the hybrid FB‐CB strategy is found to be the preferred strategy for all company scales. This paper demonstrates how the simulation framework acts as a valuable test bed for assessing the potential of novel continuous strategies to cope with different scales of operation and decisional drivers.

The analysis demonstrates scenarios where continuous processing can offer COG and operational robustness benefits. Industrial uptake of continuous technologies will also be affected by further considerations that include the the development effort required, technology readiness for large‐scale manufacture (e.g., availability of GMP skids, online analytics and control, hardware reliability), protocols and training to deal with the extra operational complexity, and regulatory concerns.

## References

[1] DiMasi JA , Feldman L , Seckler A , Wilson A. Trends in risks associated with new drug development: success rates for investigational drugs. Clin Pharmacol Therap. 2010;87:272–277. 20130567 10.1038/clpt.2009.295

[2] Farid SS. Process economics drivers in industrial monoclonal antibody manufacture In: Gottschalk U, editor. Process Scale Purification of Antibodies: Hoboken, NJ, USA: John Wiley & Sons, Inc; 2009;239–262.

[3] Morgan S , Grootendorst P , Lexchin J , Cunningham C , Greyson D. The cost of drug development: a systematic review. Health Policy. 2011;100:4–17. 21256615 10.1016/j.healthpol.2010.12.002

[4] Paul SM , Mytelka DS , Dunwiddie CT , Persinger CC , Munos BH , Lindborg SR , Schacht AL. How to improve R&D productivity: the pharmaceutical industry's grand challenge. Nat Rev Drug Discov. 2010;9:203–214. 20168317 10.1038/nrd3078

[5] Bogdan B , Villiger R. 2010. Valuation in Life Sciences. Valuation in Life Sciences: A Practical Guide, Third Edition:67–303.

[6] Nie W. Cost evaluation and portfolio management optimization for biopharmaceutical product development. PhD Thesis. University College London; 2015.

[7] Kelley BD. Very large scale monoclonal antibody purification: the case for conventional unit operations. Biotechnol Prog. 2007;23:995–1008. 17887772 10.1021/bp070117s

[8] Kelley B. Industrialization of mAb production technology: the bioprocessing industry at a crossroads. mAbs. 2009;1:443–452. 20065641 10.4161/mabs.1.5.9448 PMC2759494

[9] Farid SS , Pollock J , Ho SV. Evaluating the economic and operational feasibility of continuous processes for monoclonal antibodies In: Subramanian G, editor. Continuous Processing in Pharmaceutical Manufacturing. First Edition Weinheim, Germany: Wiley‐VCH Verlag GmbH & Co. KGaA; 2015; Ch 17. doi:10.1002/9783527673681.ch17.

[10] Croughan MS , Konstantinov KB , Cooney C. The future of industrial bioprocessing: Batch or continuous? Biotechnol. Bioeng. 2015;112:648–651. doi:10.1002/bit.25529 25694022 10.1002/bit.25529

[11] Mahajan E , George A , Wolk B. Improving affinity chromatography resin efficiency using semi‐continuous chromatography. J Chromatogr A. 2012;1227:154–162. 22265178 10.1016/j.chroma.2011.12.106

[12] Warikoo V , Godawat R , Brower K , Jain S , Cummings D , Simons E , Johnson T , Walther J , Yu M , Wright B , McLarty J , Karey KP , Hwang C , Zhou W , Riske F , Konstantinov K. Integrated continuous production of recombinant therapeutic proteins. Biotechnol Bioeng. 2012;109:3018–3029. 22729761 10.1002/bit.24584

[13] Godawat R , Brower K , Jain S , Konstantinov K , Riske F , Warikoo V. Periodic counter‐current chromatography—design and operational considerations for integrated and continuous purification of proteins. Biotechnol J. 2012;7:1496–1508. 23070975 10.1002/biot.201200068

[14] Bisschops M , Brower M. The impact of continuous multicolumn chromatography on biomanufacturing efficiency. Pharm. Bioprocess. 2013;1:361–372.

[15] FDA. 2011 Advancing regulatory science at FDA—A strategic plan. August. http://www.fda.gov/downloads/ScienceResearch/SpecialTopics/RegulatoryScience/UCM268225.pdf (last accessed 18 August 2014)

[16] Pollock J , Ho SV , Farid SS. Fed‐batch and perfusion culture processes: economic, environmental, and operational feasibility under uncertainty. Biotechnol Bioeng. 2013;110:206–219. 22806692 10.1002/bit.24608

[17] Pollock J , Bolton G , Coffman J , Ho SV , Bracewell DG , Farid SS. Optimising the design and operation of semi‐continuous affinity chromatography for clinical and commercial manufacture. J Chromatogr A. 2013;1284:17–27. 23453463 10.1016/j.chroma.2013.01.082

[18] Farid SS , Thompson B , Davidson A. Continuous Bioprocessing: The real thing this time?. mAbs. 2014;6:1357–1361. doi:10.4161/mabs.36151. 25484060 10.4161/mabs.36151 PMC5155669

[19] Farid SS. Established bioprocesses for producing antibodies as a basis for future planning. Cell Cult Eng. 2006;101:1–42. 10.1007/10_014 16989256

[20] Marichal‐Gallardo PA , Álvarez MM. State‐of‐the‐art in downstream processing of monoclonal antibodies: process trends in design and validation. Biotechnol Progress. 2012;28:899–916. 10.1002/btpr.1567 22641473

[21] Lim AC , Zhou YH , Washbrook J , Sinclair A , Fish B , Francis R , Titchener‐Hooker NJ , Farid SS. Application of a decision‐support tool to assess pooling strategies in perfusion culture processes under uncertainty. Biotechnol. Progr. 2005;21:1231–1242. 10.1021/bp049578t 16080707

[22] Lim AC , Washbrook J , Titchener‐Hooker NJ , Farid SS. A computer‐aided approach to compare the production economics of fed‐batch and perfusion culture under uncertainty. Biotechnol Bioeng. 2006;93:687–697. 16259001 10.1002/bit.20757

[23] Wojciechowski PW , Smit HI , Myers MM , Voronko PJ , Laverty T , Ramelmeier RA , Siegel RC. Making changes to a biopharmaceutical process during development and commercial manufacturing: The REMICADE story In: Shukla AA, Etzel MR, Gadam S, editors. Process Scale Bioseperations for the Biophamceutical Industry. London, UK: Taylor Francis Group; 2007:507–523.

[24] Shevitz J. Fluid Filtration System patent 6544424; 2008.

[25] Crowley J , Wubben M , Coco Martin JM. Process For Cell Culturing by Continuous Perfusion and Alternating Tangential Flow patent 20080131934; 2008.

[26] Gagnon P. Technology trends in antibody purification. J Chromatogr A. 2012;1221:57–70. 22071423 10.1016/j.chroma.2011.10.034

[27] Materie, J. Advances in continuous chromatography: Increasing the productivity of the MAb direct capture step using 3‐column—periodic counter current chromatography. 239th ACS National Meeting, San Francisco, CA. March 21–25; 2010.

[28] Miao, F , Jen D , Jones T , Vora H , Ramelmeier A , Lacki KM , Bryntesson LM . A feasibility study of periodic counter‐current chromatography for the capture of monoclonal antibodies from crude harvest with MabSelect protein A resin. GAb, Nice, France; 2004.

[29] Daszkowski, T. Continuous processing in biotech production as an alternative to a modern batch, single‐use facility. ECI Integrated Continuous Biomanufacturing, Barcelona, Spain. October 20–24; 2013.

[30] Bisschops M , Frick L , Fulton S , Ransohoff T. Single‐use, continuous‐countercurrent, multicolumn chromatography. BioProcess Int. 2009;7:S18–S23.

[31] Lyle, S. Continuous protein A chromatography for the purification of monoclonal antibodies using a three‐column system. IBC Antibodies, Carlsbad, CA; 2010.

[32] Farid SS , Washbrook J , Titchener‐Hooker NJ. Modelling biopharmaceutical manufacture: design and implementation of SimBiopharma. Comput Chem Eng. 2007;31:1141–1158.

[33] Sheldon RA. The E factor: fifteen years on. Green Chem. 2007;9:1273–1283.

[34] Hwang CL , Yoon K. Multiple Attribute Decision Making—Methods and Applications. A State‐of‐the‐Art Survey. Lecture Notes in Economics and Mathematical Systems. Berlin, Heidelberg, New York: Springer Verlag; 1981.

[35] Farid SS , Washbrook J , Titchener‐Hooker NJ. Combining multiple quantitative and qualitative goals when assessing biomanufacturing strategies under uncertainty. Biotechnol Progr. 2005;21:1183–1191. doi 10.1021/bp050070f 10.1021/bp050070f 16080700

[36] George E , Titchener‐Hooker NJ , Farid SS. A multi‐criteria decision‐making framework for the selection of strategies for acquiring biopharmaceutical manufacturing capacity. Comput Chem Eng. 2007;31:889–901. doi 10.1016/j.compchemeng.2006.12.009

[37] Deb K. 2008 Multi‐objective Optimization using Evolutionary Algorithms. Chichester: John Wiley & Sons, Ltd.

[38] Triantaphyllou E. Multi‐Criteria Decision Making Methods: A Comparative Study. Netherlands: Kluwer Acadmic Publishers; 2000.

[39] Leigh SR. Evolution of human growth spurts. Am J Phys Anthropol. 1996;101:455–474. 9016361 10.1002/(SICI)1096-8644(199612)101:4<455::AID-AJPA2>3.0.CO;2-V

[40] Chapman K , Pullen N , Coney L , Dempster M , Andrews L , Bajramovic J , Baldrick P , Buckley L , Jacobs A , Hale G , Green C , Ragan I , Robinson V . Preclinical development of monoclonal antibodies Considerations for the use of non‐human primates. mAbs. 2009;1:505–516. 20065651 10.4161/mabs.1.5.9676 PMC2759500

[41] Ogden CL , Fryar CD , Carroll MD , Flegal KM. Mean body weight, and body mass index, United States 1960–2002. In: Surveys DoHaNE, editor. CDC; 2004. 15544194

[42] Kelley BD , Tobler SA , Brown P , Coffman JL , Godavarti R , Iskra T , Switzer M , Vunnum S. Weak partitioning chromatography for anion exchange purification of monoclonal antibodies. Biotechnol Bioeng. 2008;101:553–566. 18727127 10.1002/bit.21923

[43] Ho SV , McLaughlin JM , Pollock J , Farid SS. Toward Greener Therapeutic Proteins In: Tao J, Kazlazuskas R, editors. Biocatalysis for Green Chemistry and Chemical Process Development. New Jersey: John Wiley & Sons; 2011:197–219.

